# Durum Wheat Kernel: Influence of the Genotype and Environment on the Mineral Profile of Grains and Ashes

**DOI:** 10.3390/plants14223414

**Published:** 2025-11-07

**Authors:** José Moreira, Sara Rodrigo, Nuno Pinheiro, Rita Costa, Armindo Costa, José Dôres, Manuel Patanita, Benvindo Maçãs, Roberta Leitão, Mauro Guerra, Ana Sofia Bagulho

**Affiliations:** 1 Instituto Nacional de Investigação Agrária e Veterinária, I.P.—Elvas, Estrada de Gil Vaz Ap. 6, 7350-901 Elvas, Portugal; zeecmoreira@hotmail.com (J.M.); saramrodrigo@gmail.com (S.R.); nuno.pinheiro@iniav.pt (N.P.); rita.costa@iniav.pt (R.C.); armindo.costa@iniav.pt (A.C.); benvindo.macas@iniav.pt (B.M.); 2 Instituto de Investigación de la Dehesa (INDEHESA), Universidad de Extremadura, Avda. de Elvas, 06006 Badajoz, Spain; 3 GeoBiotec—GeoBioSciences, GeoTechnologies and GeoEngineering Research Center, NOVA School of Sciences and Technology, NOVA University Lisbon, Campus da Caparica, 2829-516 Caparica, Portugal; mpatanita@ipbeja.pt; 4 I.P. Beja/ESA—Instituto Politécnico de Beja, Escola Superior Agrária, Departamento de Biociências, Rua Pedro Soares, 7800-295 Beja, Portugal; jdores@ipbeja.pt; 5 GREEN-IT Bioresources for Sustainability, ITQB NOVA, Av. da República, 2780-157 Oeiras, Portugal; 6 LIBPhys, LA-REAL, NOVA School of Sciences and Technology, NOVA University Lisbon, Campus da Caparica, 2829-516 Caparica, Portugal; betagleitao@gmail.com (R.L.); mguerra@fct.unl.pt (M.G.)

**Keywords:** ash content, durum wheat quality, mineral composition, semolina yield

## Abstract

Thirteen genotypes of durum wheat were grown in two different environments in Portugal. Grain and ash mineral profile, as well as protein content, test weight, and grain ash content were evaluated. Genotype, environment, and their interaction explains the variation in the quality traits, with the environment having the highest influence. Mineral profile analysis was performed by the μ-EDXRF system: macroelements (K, P, Ca, Cl, and S) represented 99% of the total concentration detected in the grain samples, while microelements represented up to 2% of the total concentration when analyzing the ash samples (Fe, Mn, Zn, Cu, Si, Rb, Sr, and Ti). Almost every element found in the grain and ash analysis was affected by the environment. Only K and Ca in the grain had higher concentrations in the environment with water scarcity, while the concentrations of all the detected elements except for Si and Sr were higher in the ashes in this environment. Regarding the genotype, P, S, and Cu grain concentrations were not affected by the environment. The highest grain mineral concentration was found for Gingão, suggesting a better mineral uptake and/or translocation-to-grain capacity. However, regarding the technological quality, most of the genotypes presented ash content values above the maximum specified threshold.

## 1. Introduction

Wheat is considered one of the most important cereals in the world, playing a prominent socio-economic and nutritional role in human food (it feeds >30% of the human population) [1]. Durum wheat (*Tritticum durum* Desf.) is used as a suitable raw material for the manufacture of various products such as pasta, which stands out in Europe and North America due to its widespread consumption.

In Portugal, wheat remains an important cereal for traditional farming systems in the south of the country. However, unpredictable weather situations highly influence crop yields, mainly due to the shortage of water and extreme temperatures that, at certain times of the cycle, are the most limiting factors for wheat production [2]. These conditions of water scarcity and extreme temperatures are critical depending on the phenological stage of the crop, incurring greater losses of yield and quality when they occur in late-vegetative and grain-filling periods [3]. Temperature induces a series of physiological changes in the accumulation of reserves in the grain, which interfere with all processes including the assimilation and deposition of starch, proteins, and minerals in the grain (influencing their content and composition) [4]. Water scarcity also affects photosynthesis processes, metabolites formation and remobilization, and reduces leaf area and aboveground biomass which minimizes water consumption, also causing an increase in root distribution in soil layers to enhance water and nutrient absorption [5]. The reduction in the production of photoassimilates can stimulate the remobilization of reserves for grains or reduce their accumulation, depending on the time of occurrence.

Despite the instability of the Mediterranean climate of this region, the quality of durum wheat for semolina and pasta production tends to be favored by this moderately dry climate, with a high number of hours of sunlight during the grain-filling period [6]. The quality of durum wheat is defined according to technological parameters determined at the kernel level, which are related to yield (test weight, vitreousness, and ash content), processing (protein content), and characteristics required for the final product [7]. Test weight (mass per hectoliter) and ash content are the most sensitive parameters of wheat produced in Portugal and are often outside of industry-specified ranges. In this sense, test weight is largely affected by stresses that occur during the grain-filling period. Regarding grain ash content, it is known that it is related to mineral composition, but the nature of this parameter still remains unknown.

Minerals are present in a small proportion of the wheat grain, and their presence is lower in the endosperm than in the outer layers of the grain [8]. When semolina is produced, these outer layers are removed, and a considerable part of minerals are lost [9]. Nevertheless, legal restrictions have established the maximum threshold for ash content to be 0.9% DM (dry matter) for semolina suitable for pasta manufacture (Portaria No. 254/2003). This fact imposes limitations on grain ash content, assuming that it should be lower than 1.9% DM to ensure a good semolina yield and avoid the appearance of defects in pasta (i.e., black dots) [7].

Several genetic factors may influence the rate of mineral deposition in grains which can interfere with mineral root uptake ability, translocation, redistribution within the plant tissues, and remobilization to the grain [10]. The concentration of minerals in the grain changes on a specific basis for each element, and the proportion of each element in the morphological section of the grain depends on the genotype, though environmental factors also play a role [8,9,11]. Other genetic aspects that affect the distribution of minerals in the grain are shape, texture, and dimension of the grain: larger grains are associated with lower relative concentrations of nutrients, even if the total amount per kernel is not affected [12].

Edaphoclimatic conditions (soil type, nutrient availability, pH, water holding capacity, insolation, and temperature, among others) as well as agronomic management (sowing density, crop rotation, fertilization, etc.) and physiological aspects (stage of grain maturation at harvest, plant health) can also influence the mineral content in wheat grain [13,14].

Mineral compounds such as potassium phosphates and sulfates or calcium and magnesium compounds are very common in wheat grain [15]; in fact, Meenu et al. [16] indicated in their research that the most abundant minerals of wheat grain are phosphorus, potassium, magnesium, and calcium. According to Grant et al. [17], durum wheat grain is characterized by a low sodium content (0.01–0.05 mg/g), while the typical concentrations of the other macroelements are 3.8–5.5 mg/g for potassium (K), 1.8–5.2 mg/g for phosphorus (P), 1.0–1.5 mg/g for magnesium (Mg), and 0.32–0.47 mg/g for calcium (Ca). Copper (Cu), iron (Fe), manganese (Mn), selenium (Se), and zinc (Zn) are also found in kernels but in lower concentrations. According to Emam et al. [18], wheat grain is a source of essential minerals for the global population due to the high consumption of wheat products throughout the world. Considering that above two billion people worldwide are affected by mineral deficiencies, especially micronutrients [19], the increase in minerals in wheat kernel could contribute to improving human health. Thus, many biofortification examples are found in the literature, where researchers obtained an increase in some mineral elements, critical for human health, such as Zn, Se, I, or Fe, which are proven to improve wheat consumers health, whether animals or human beings [20,21,22]. However, researchers should not ignore the maximum ash content threshold set by national regulations, even when grain ash content does not influence nutritional quality, it can influence technological quality, which is not desirable for the durum wheat product industry.

In this experiment, 13 genotypes of durum wheat were grown in two different environments, and the effect of genotype and environment on the main grain quality traits (protein, test weight and ash content) and the mineral profile in grain and ashes were evaluated, with the aim of determining the influence of each factor on every element detected in the analysis. Thus, although this study was conducted in the south of Portugal, its main conclusions can be extrapolated and applied to other Southern Mediterranean regions.

## 2. Materials and Methods

### 2.1. Plant Material

The wheat genotypes tested in this experiment (*Tritticum durum* Desf.) include thirteen commercial genotypes commonly used in Portugal sourced from Spain (Arcoduro, Don Norman, Don Ricardo, Trimulato), Italy (Massimo Meridio, Antalis, Kenobi, Aventadur), and Portugal (Celta, Fado, Vadio, Bridão, Gingão). Portuguese genotypes were obtained from the cereal breeding program of INIAV, I.P. Genotype data description (origin, growth habit, and registration year) is presented in Supplementary Material Table S1.

### 2.2. Experimental Site

Field experiments were conducted in two different locations with different environmental conditions: −Alto Alentejo (AA) (38°53′39″ N, 7°03′20″ W) and −Baixo Alentejo (BA) (38°02′14″ N, 7°53′06″ W), representing the most important provinces in Portugal for cultivating durum wheat crop.

These two soils present some differences: AA soil, belonging to the fluvisols group, showed a high level of mineral nutrients and good drainage capacity; BA soil, belonging to the vertisols group, is dark colored and characterized by well-defined layers of calcium carbonate, high level of vegetable nutrients, and a clayey to very clayed texture [23].

### 2.3. Field Experiment

Experiments in each environment were arranged in a randomized complete block design with three replicates, with an elemental plot area of 9.6 m^2^. Sowing took place on 12th December and harvesting on 5th–6th July. Conventional fertilization of 165 UN fractionated throughout the culture cycle (one basal fertilization and three top-dressed fertilizations) were performed, and herbicide protection (post-emergence) and antifungal treatments were applied (in stem elongation -GS30-GS33- and booting -GS41-GS47).

### 2.4. Quality Evaluation

Grain test weight and protein content were evaluated by NIR Infratec 1241 (FOSS, Hillerød, Denmark), according to BS EN 15948: 2020 [24].

Aliquots of any sample were ground in a Cyclotec 1093 mill with a sieve of 0.5 mm (Tecator, Hillerød, Denmark), and ash content was determined after a 900 °C-incineration in a Nabertherm laboratory oven (LT 15/11, Lilienthal, Germany), following the official rule ISO 2171:2023 [25]. Grain mineral characterization (GR), as well as grain ash characterization (GRash) were developed by means of μ-EDXRF (Micro energy dispersive X-ray fluorescence) [26] comprising a benchtop spectrometer M4 Tornado (Bruker, Berlim, Germany), with a X-ray tube cooled by air, micro-focus side window Rh tube, and powered by a low-power HV generator. The detection of fluorescence radiation was performed by an energy dispersive silicon drift detector with 30 mm^2^ sensitive area. The X-ray generator was operated at 50 kV and 100 μA without the use of filters and in vacuum in order to improve the ionization of elements of low atomic number.

Quantification of elements was performed based on the fundamental parameter method for bulk samples, where they were previously compressed (10 ton, 2 min) and glued onto a mylar sheet mounted on slide frames (50 × 50 mm). The glue was composed of light elements which cannot be detected.

### 2.5. Statistical Analyses

Statistical analyses were performed using the statistical package IBM SPSS software (version 20, IBMCorp., Armonk, NY, USA) Statistics. Analysis of variance (ANOVA) was determined with the general linear model procedure to study the effect of each genotype, environment, and their interaction with quality parameters and mineral composition. Means were compared using Tukey Student’s test (significance level *p* < 0.05). The relationship between ash content and mineral elements was examined by Pearson correlation coefficients using the results from the two locations, and the results were recognized as statistically significant at *p* ≤ 0.05.

## 3. Results

### 3.1. Climatic Conditions

**Figure 1:**
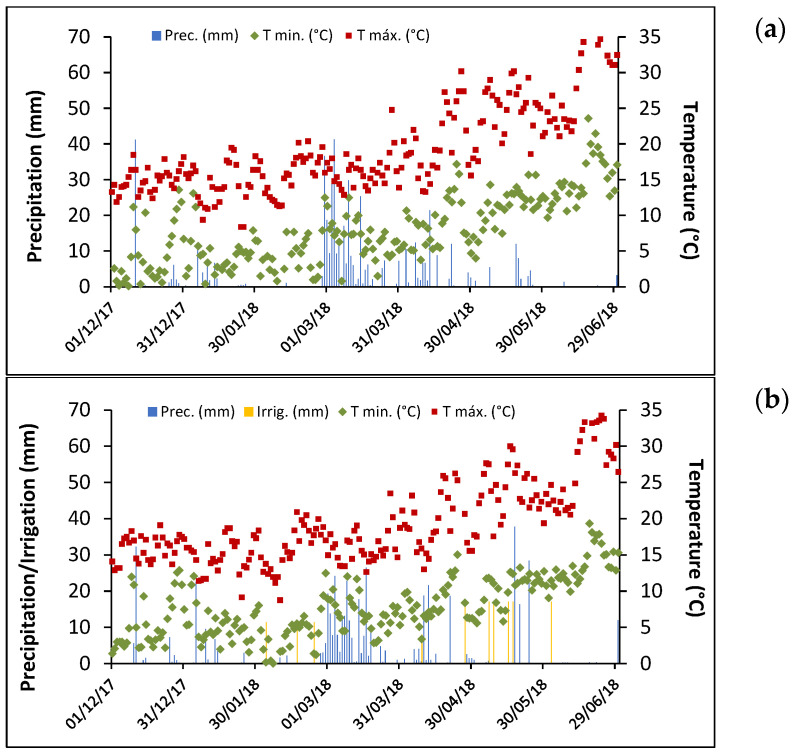
Climatic conditions at Alto Alentejo (**a**) and at Baixo Alentejo (**b**): daily record of rainfall (Prec.), irrigation (Irrig.), maximum daily temperature (Tmáx.), and minimum daily temperature (Tmin.). Data provided by meteorological stations at experimental sites.

Both sites of the experiments, AA and BA, are located in two subregions of Alentejo, the main cereal-producing region in Portugal, where climate is usually characterized by Mediterranean conditions with high irregularity in both rainfall and temperatures across seasons and years. Climatic conditions at both sites during the field experiments are presented in Figure 1.

The temperatures at the two sites were quite similar in almost every month, showing a parallel average of maximum temperatures (around 15–16 °C) along winter months (December, January, February) and with an average of minimum temperatures of around 4–5 °C. However, BA showed minimum temperatures below zero in January, which coincides with the beginning of tillering, delaying or lengthening this phenological phase. In the spring months (March, April, May), the average of maximum temperatures was 16 °C in March, 19–20 °C in April, and 24–25 °C in May, but again, some differences were found between the two locations: AA showed higher minimum and maximum temperatures during the grain-filling period which significantly modified the grain-filling capacity of the plants. Thus, the number of days with maximum temperatures above 25 °C was greater in AA (6 days in late April and 16 in May) than in BA (10 days in May). Total rainfall was very similar in both sites while the experiments took place, with a total amount of 519 mm in AA and 518 mm in BA. However, winter precipitation was 20 mm higher in AA than in BA, mainly concentrated on the first few days of January. Spring total rainfall amount was more or less the same for both sites, but again, the distribution was slightly different, given that in the rainy month (March), in which the monthly rainfall in AA was 30 mm higher than in BA, there was better rainfall distribution in BA. Moreover, to increase the differences in management, in BA, irrigation took place when necessary (February—34 mm, April—25 mm, May—68 mm and June—17 mm), preventing plants from suffering from water stress, which definitely happened in AA.

### 3.2. Genotype and Environmental (Site) Effects on Durum Wheat Technological Quality

Genotype and environment contributed for variation in the three quality traits (test weight, protein and ash content), with greater importance of environment, and also, with a significant effect for the interaction between both (Table 1).

**Figure 2:**
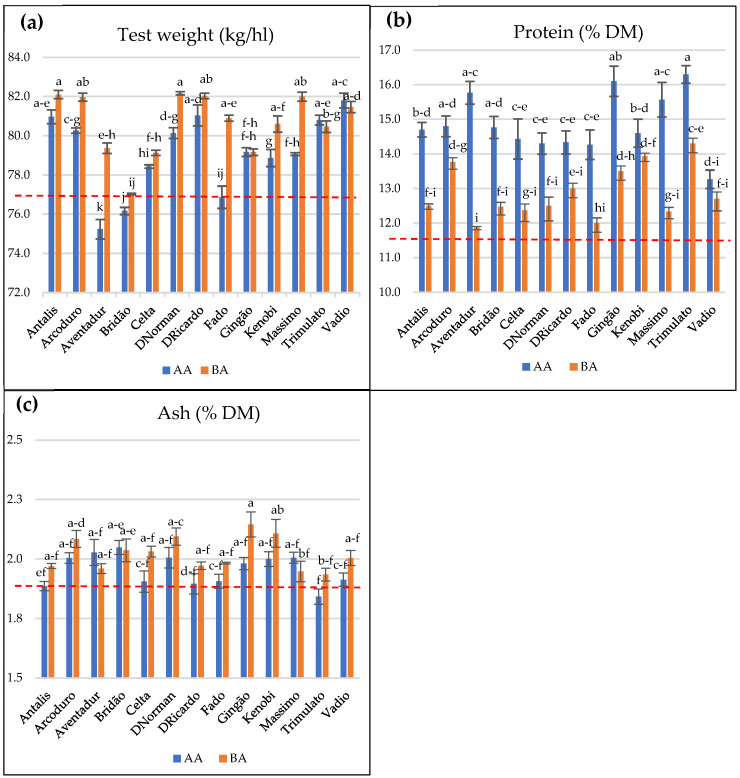
Mean values (n = 3) ± standard error of (**a**) test weight, (**b**) protein content, (**c**) ash content for 13 durum wheat genotypes grown in Alto Alentejo (AA) and Baixo Alentejo (BA) experiments. Different letters indicate significant differences between the genotype × environment combinations, according to Tukey test (*p* ≤ 0.05). Red line indicates the industrial acceptable specification of each parameter.

Regarding the test weight, Figure 2a showed better results for BA than AA in most genotypes, with BA data being consistently above the minimum industrial reference value of 77 kg/hL. In the case of AA, only Aventadur and Bridão genotypes did not reach the given reference value. Conversely, protein data for AA showed values between 13.3% DM and 16.3% DM, which means that every value was at least about 2% above the minimum industrial reference value of 11.5% DM for durum wheat, while the protein results for BA did not reach 14.5%, even for the best results, being below 13.5% DM most of the time (Figure 2b). Finally, regarding the ash content (Figure 2c), almost all combinations of environment x cultivar exceeded the maximum reference value for industrial purposes in durum wheat (1.9% DM), with the exception of genotype Trimulato grown in AA.

### 3.3. Genotype and Environmental (Local) Effects on Durum Wheat Grain and Ash Mineral Composition

Before analyzing the numerical data of the different elements, it is necessary to indicate that macroelements represented about 99% of the total concentration detected in the grain samples (GR samples) of the two environments, while microelements represented about 1% (Table 2). Calcium (Ca), chlorine (Cl), potassium (K), phosphorus (P), and sulfur (S) were the five macroelements detected by μ-EDXRF (all with higher atomic number than aluminum).

In both environments, K was the most abundant element, followed by P, Ca, and S in the same order of magnitude, and lastly, Cl. According to Gallardo et al. [26], magnesium (Mg) and sodium (Na) could not be detected accurately by μ-EDXRF because the energy (<3 keV) involved is low, which is often reabsorbed by the sample or blocked by the medium between the sample and the detector (typically a beryllium window). In fact, this can occur with elements with lower atomic number than aluminum.

Regarding microelements, copper (Cu), iron (Fe), manganese (Mn), and zinc (Zn) were the four microelements detected in the grain samples of both locations (Table 2), with Fe being the most abundant microelement, followed by Mn, Zn, and Cu.

After comparing the two environments, significant differences in grain mineral content were found: AA had a higher amount of K and Ca, while the contents of P, S, Cl, Zn, and Cu were higher in BA grains. No significant differences were observed between the iron and manganese grain contents of the two environments (Table 2).

The analysis of the ash samples detected three macroelements (Ca, K, and P) and eight microelements: Cu, Fe, Mn, and Zn as in the grain samples, as well as Rb, Si, Sr and Ti (Table 2). However, Cl and S were not detected in the ash samples. As expected, the elements found in higher concentrations in ashes were K, P, and Ca, in this order. The microelement concentration increased to about 2% of the total detected in grain ash samples, but the concentration of macroelements still remained comparatively higher (98%).

The results of the ANOVA for the macro- and microelement concentrations in grain (GR) and grain ash (GRash) samples are presented in Table 3. Genotype (G) and environment (E) contributed to the variation in most macro- and microelements detected in grain samples, with greater influence by the environment for all the elements excluding Fe and Mn, which were determined to not be affected by the environment.

The genotype did not affect Cu, S, and P grain concentrations, and a small significant effect was found for the interaction G × E in the concentrations of Fe, K, P, and Zn (Table 3). The genotypes behaved differently in terms of the grain mineral profile for the two environments, showing a great effect of edaphoclimatic conditions in the uptake or translocation of the minerals in the plant, which may be due to the different availability of minerals in soil due to water availability.

Grain ash sample results revealed that all the detected elements were significantly influenced by the genotype and environment, except for P and Rb that were not affected by E or G, respectively. The interaction between the two factors significantly influenced the amount of almost all the elements in grain ash samples, except for Mn, Rb, Si, and Ti.

The concentrations of grain macro- and microelements (GR samples) affected by the genotype × environment interaction are presented in Table 4.

It is important to highlight that within each environment (AA or BA), no significant differences were found among the genotypes regarding P or Zn data. Concerning K concentration, only Fado (9.43 g/kg) was significantly different from Don Ricardo (7.06 g/kg) in environment AA. Similarly, for Fe concentration, only Fado (51.8 mg/kg) was significantly different from Celta (34.9 mg/kg) in environment AA. When comparing environments by genotype, K values in most of the genotypes in AA were significantly higher than those obtained in BA, except for K concentrations of Celta and Don Ricardo, with no significant differences between environments. Conversely, in the case of P results, only Celta and Don Ricardo were found to present significant differences between environments, showing higher data in BA than in AA (2.29 and 2.23 g/kg for Celta and Don Ricardo in BA, respectively, vs. 1.41 and 1.40 g/kg for Celta and Don Ricardo in AA, respectively). Similarly, when significant differences were found between environments by each genotype regarding Zn grain concentration, values were higher in BA than in AA (as similarly observed for Antalis, Aventadur, Celta, Don Norman, Don Ricardo, Kenobi, and Vadio genotypes). Finally, it can be observed in Table 4 that Fe concentration in grain is barely affected by the two factors (G and E); thus, only Fado genotype grown in AA was found to show significantly higher Fe concentration (51.8 mg/kg) than Celta grown in AA (34.9 mg/kg) and Massimo Meridio and Bridão genotypes grown in BA (34.4 and 32.8 mg/kg, respectively).

Elements found in grain such as Ca, Cl, Cu, Mn, and S were not affected by the interaction of the factors (G × E). Table 5 indicates that Ca results in AA were higher than in BA, contrary to what happened with Cl, Cu, and S, of which data in BA were found to be higher than in AA. Regarding Ca and Cl analysis by genotype (Table 5), Gingão showed higher values than the rest of the genotypes in any environment (0.700 g/kg in AA and 0.524 g/kg in BA for Ca; 0.264 g/kg in AA and 0.336 g/kg in BA for Cl), while Kenobi in BA seemed to accumulate a higher amount of Cu (10.27 mg/kg) and S (0.676 g/kg) in its grain.

With reference to Mn, for both environments, the Trimulato genotype was situated in the group of genotypes with higher grain concentration (48.1 mg/kg in AA and 46.3 mg/kg in BA). Notably, Don Ricardo, which was found to be among the genotypes accumulating less Ca and Cl in the grains for the two environments and less Mn, P, and Zn in the AA environment (Table 4 and Table 5). In this case, genetics seemed to be the reason behind low mineral accumulation.

The results of the analysis of the concentration data for macro- and microelements of grain ashes (GRash samples) affected by the genotype × environment interaction are presented in Table 6.

Here it can be seen that the trends observed in the GR data for K, P, and Zn are similar to those obtained in GRash, showing a higher amount of K in samples coming from AA (169.6–215.8 g/kg) than BA (80.8–128.7 g/kg), and similar P and Zn results in both environments for most genotypes (excluding Arcoduro). This similar trend in both analysis (mineral in GR and in GRash) is supported by the correlations found in this work between ash content and grain element concentrations (Table 7).

In Table 7, it can be seen that almost every element detected in the grain (except for Ca, Fe, and Mn) showed correlation, whether positive or negative, with the grain ash content. In the case of Fe, while in GR almost no significant differences were found, in GRash most of the genotypes showed a higher concentration of this mineral in AA than in BA, with values ranging between 0.707 and 1.658 g/kg.

Regarding Ca data (Table 6), most of the genotypes showed significantly higher values of this mineral in AA than in BA, with 11.4 g/kg being the lower value in AA and not exceeding 11.2 g/kg in BA, and neither Fado, Massimo Meridio, nor Vadio showed significant differences between environments. Copper results did not show important statistical differences between environments, only Arcoduro and Kenobi Cu data were higher in AA than in BA (0.176 g/kg and 0.101 g/kg for Arcoduro, 0.202 g/kg and 0.143 g/kg for Kenobi, respectively); thus, Kenobi genotype in AA (0.202 g/kg) showed higher Cu concentration than Aventadur (0.133 g/kg), Celta (0.126 g/kg), Don Norman (0.135 g/kg), Fado (0.121 g/kg), Massimo Meridio (0.127 g/kg), and Vadio (0.121 g/kg) in the same environment. Although Sr ash content was affected by the environment (Table 3), with a higher mean value in BA than in AA, when comparing each genotype between the two environments, it was verified that these differences were not significant. Comparing by genotypes, Gingão was found to be a Sr-accumulator genotype, showing higher Sr concentration in the grain ashes than genotypes Arcoduro, Bridão, Don Ricardo, Fado, and Vadio, no matter which environment they were grown at.

With reference to the last minerals detected in GRash, they were only affected by the main factors and not by the G × E interaction, data are presented in Supplementary Material (Table S3). Notably, Trimulato and Gingão showed the highest Mn concentrations in both environments, while Arcoduro genotype seemed to accumulate less amounts of Rb and Si in BA than the rest of the genotypes.

## 4. Discussion

It is widely known that temperature and rainfall play crucial roles in wheat crop development, severely affecting not only yield [27,28,29] but also technological quality. In the current study, the higher soil water availability in BA during the plant cycle, thanks to the use of irrigation in critical periods, together with the higher number of days with maximum temperatures above 25 °C occurring during the grain-filling period in AA seem to be the responsible for the significant environmental influences. This is aligned with other studies such as by Campiglia [30], who found that a shortage of soil water availability in critical periods, combined with high temperatures in spring, significantly reduced grain yield and quality, based mainly on protein, gluten, or ash content. Thus, the test weight in our study was shown to be significantly higher in BA than in AA (Figure 2a), due to both a shortage in water availability and high temperatures in spring. However, it is necessary to highlight that some of the genotypes did not show statistical differences between sites regarding test weight results, suggesting that breeding for climate adaptation is a great way to avoid yield reductions caused by weather conditions. Conversely, nine out of the thirteen genotypes tested in this study presented significantly higher grain protein content in the drier environment (AA), compared to the one with irrigation (BA); as Michel et al. [31] explained in their research, this could be explained by the lower amount of starch in the grain due to the reduction in the photosynthesis efficiency and, hence, the lower dilution of other grain components such as protein.

Concerning ash content, minor differences were found for genotype, but significant differences were revealed when considering the two different sites. This is aligned with the conclusion obtained by Chaurand et al. [32], who also found differences between environments, especially regarding water and nitrogen availability. Results from our research revealed higher grain ash content in the environment with no water scarcity, BA, contrary to the results found in France by Chaurand et al. [32] but similar to the results of Miravalles [33], who related low precipitation during crop cycle with a lower amount of ash content; this could be due to the possibility of minerals being dissolved in water at a higher extent and, thus, increasing availability for plant uptake, as stated by Adams et al. [34].

According to Von Grebmer [35], more than 2 billion people across the globe suffer from one or more micronutrient malnutrition, which significantly affects physical and mental development, immunity, and overall health. To avoid this, improving nutrient levels in the edible parts of staple crops could be a viable cost-effective and sustainable approach; thus, cultivating crops in the most favorable agronomical conditions would help to reduce the burden of micronutrient deficiencies in the world [36].

According to FAO (www.fao.org), wheat is one of the staple foods worldwide, so the mineral profile of wheat kernel is becoming a great concern in recent years, not only attracting the interest of scientists, but also governments or general society, who are increasingly concerned about the influence of food quality (especially essential nutrients) on human health, which is reflected in a country’s healthcare costs. Our work attempts to determine the influence of the environment and the genotype on wheat plant mineral uptake and translocation, to finally determine the mineral profile grain wheat. In our research, K, P, and Ca were the minerals present in the greatest amount in wheat kernel, which is aligned with other findings [8,16]. These authors also refer to Mg but this element could not be detected in this study, probably due to the use of different methodologies in the analysis. All these elements are considered essential for human life; thus, K was recently associated with a reduction in cardiovascular events [37], P disorders seem to impact bones or soft tissues as well as kidney function, a deficient intake of Mg implies insulin resistance or headaches, among others, and Ca deficiencies affect not only bones but also muscles and nerves [38].

In this sense, the accumulation rate of minerals and the influence of climatic conditions on it for the most important minerals for human health are revealed as a key matter nowadays to improve population health. Therefore, it is interesting to highlight that K, which is the nutrient most accumulated before anthesis, with little or no accumulation after anthesis, regardless of seasonal conditions, contributes proportionally much more to the content of a mature grain than P [39], as can be seen in our data (Table 2). The concentration of K found in our work (>8 g/kg) was slightly higher than that found by Grant et al. [17] (3.8–5.5 g/kg) or by El Houssni et al. [40] (4.1–5.1 g/kg), probably due to a lower water availability in our conditions. Water scarcity, as stated above, can affect test weight, lowering it, and causing a lower starch content, which can increase K (mineral) concentration [41]. However, a severe shortage of water availability during the growth cycle can cause plant stress, which seriously affects the availability of macro- and microelements needed for plants [42]. Thus, the total amount of rainfall is not the most important parameter to determine the final mineral content, because, as seen in our experiment, the absence of precipitation during the end of January and the entire month of February (Figure 1), not compensated with supplementary irrigation in AA, may have contributed to a lower absorption of some minerals in this environment. This hypothesis is supported by Brier et al. [8], who observed that the greatest accumulation of nutrients occurs before anthesis for most elements, and Raza et al. [43], who found out that water deficit can reduce the absorption of phosphorus by around 62%, with these decreases being recorded in the tillering phase.

Regarding P and Ca concentrations, they were within the range of that found in the literature: 1.6–5.2 g/kg [17,40,44] for phosphorus and 0.32–0.47 g/kg for calcium [17,44]. El Houssni et al. [40] obtained lower values for calcium concentration than what we obtained in this study (0.14–0.19 g/kg), again, probably due to the significant influence of the environment exposed in our work; in fact, it is well known that mineral concentration in soil limits plant mineral uptake [45]. In our study, higher Cu, P, and Zn soil content in BA (Table S2 in Supplementary Material) can explain the statistically higher GR concentration in those minerals (Table 2) in BA environment. Synergetic effect between K and Ca, was found by Malavolta [46], which can explain the similar behavior of both elements in the AA environment, showing a higher concentration in AA than in BA, while the rest of the elements were higher in BA. Close values of Cu, Fe, and Zn have been referenced in the literature on durum wheat [9,47,48,49]. In addition, El Houssni et al. [40], obtained values of iron (average value of 41.1 mg/kg) in samples of durum wheat similar to those presented here (42.1–44.0 mg/kg), showing a stronger effect of the species (durum wheat vs. soft wheat) than the environment. This is in accordance with our results, because Fe grain concentration was affected by the genotype but not by environmental conditions (Table 3). However, other authors, who analyzed a large group of cultivars in different environments, observed effects of the environment not only in Fe concentration but also in Mn, S, and Zn concentrations [44]. Nevertheless, these authors were investigating organic farming which can affect the uptake of the minerals by roots and nutrient use efficiency. This fact could explain the higher concentrations for S (1.23 g/kg) and for Zn (36.2 mg/kg) found in their work compared to our data (0.5–0.6 g S /kg, 20–30 mg Zn/kg).

With reference to the different Ca and Zn grain concentrations found in the two studied environments in our experiments, it is worthy to highlight that, according to Racz et al. [50], a kind of antagonism between these two elements may exist, which could explain why the environment presenting higher GR concentration in Ca also presents significantly lower Zn grain concentration.

Interestingly, as can be seen in our results, not all the elements are influenced by the environment in the same way. Hussain et al. [44] concluded that the influence of genotype and environment on the grain concentration of various minerals was found to vary in relation to genotype group and mineral: environment was found to be more important than genotype for the concentration of all minerals except Se and Mo in a group of spelt, durum, and bread wheat breeding lines, while the influence of genotype was higher as compared to the environmental conditions for the grain concentration of Fe, Mg, Mn, and S. This last assessment is confirmed by our data, which did not show any statistical differences between environments for Fe or Mn grain concentrations (Table 3). Of course, as said in previous paragraphs, it is necessary to take into account the test weight influence on mineral concentration due to the dilution effect. Both test weight as well as grain protein content are highly influenced by environmental conditions [6,7,51,52,53] and this can indirectly affect the mineral concentration.

Analyzing the two locals separately, it was found that the genotypic influence on the accumulation of minerals in the grain was more evident in BA than in AA (Table 5). This could be explained by the different photosynthetic capacities and chlorophyll concentrations by genotype [44,54] that affects the general status of the plant. The assimilation and translocation of minerals is also affected by genotype, influencing compensatory mechanisms developed to deal with deficiencies in macronutrients and micronutrients in the soil or in root growth and branching that allows them to further exploit the soil [55,56].

Ash content represents the mineral content of the grain, consisting of major elements such as Ca, K, Mg, and Na, minor elements such as Al, Cu, Fe, Mn, and Zn, and traces of other elements of lesser importance. The concentration of all of them depends on the incineration conditions as well as on the original composition of the grain [57]. Ash content is widely used as a purity indicator for refinement of wheat flour and semolina because bran has approximately twenty times the ash content of the endosperm of a wheat kernel, and this explains the discrimination of bran and germ during semolina milling [58].

As predicted, the profile of minerals found in the ash samples was not exactly the same as that found in the grains: some minerals (Si, Rb, Sr, Ti) were only detected in the ashes after the concentration of mineral matter that occurs during incineration and others (Cl, S) volatilized in the same process. In the study by Johansen et al. [59] on the volatilization of mineral elements during the pyrolysis and combustion of corn straw, it was observed that 50% of chlorine (Cl) was volatilized at temperatures below 700 °C, starting below 500 °C, and around 60% of sulfur volatilization (S) occurs at temperatures below 500 °C, hence its disappearance in the ash samples.

Almost all the elements detected in the GRash samples followed the same trend as the ones in GR: they were found at a higher concentration in AA samples than in BA samples, with the exception of Si and Sr. Even though Si was not detected in GR samples, it is common that compounds containing Si are detected in wheat ash samples, as observed by Terzioglu et al. [60] who analyzed wheat ash samples and found SiO_2_, K_2_O, MgO, Fe_2_O_3_, Na_2_O, Cr_2_O_3_, MnO_2_, and CaO together with unburned carbon, water, and other residues. According to Colas et al. [61], there is no strict correspondence between the mineral composition of grain and ash, as the latter do not contain certain metallic elements and metalloids that volatilize during incineration and also contain incombustible mineral residues that come from the decomposition of organic matter. It is, therefore, natural that the correlations between ash content and grain composition are not maintained in the ash composition, which explains our results shown in Table 2, especially regarding the Cu and Zn concentrations in grain and ash. This also could explain the differences found between genotypes regarding ash composition; differences in the incineration process due to the initial mineral composition led to different final results in ash composition.

## 5. Conclusions

Wheat grain composition and quality are highly influenced by environmental conditions. Thus, water scarcity during the growth cycle favors not only a higher grain protein content but also higher Ca and K grain concentrations, while water availability implies higher durum wheat test weight, ash content, and Cl, Cu, and S concentrations. Genotype influence appears to be higher in minerals such as Fe and Mn, modulating plant mineral uptake or translocation-to-grain capacity. In conclusion, depending on the focus of the research or industry, farmers should adapt the genotype to the use and supply of water applied; Gingão genotype tends to be used when determining general mineral content, along with a controlled water scarcity to improve protein content and key minerals such as Ca and K.

## Figures and Tables

**Table 1:** Analysis of variance (MS—mean square and F value) for test weight and protein and ash contents of 13 genotypes sown at two different environments (Alto Alentejo and Baixo Alentejo). N = 78.

Source of Variation		Test Weight (R^2^ = 0.955)		Protein Content (R^2^ = 0.909)		Ash Content (R^2^ = 0.685)	
	df	MS	F	MS	F	MS	F
Model	25	11.22	43.82 ***	5.02	20.79 ***	0.016	4.53 ***
Genotype (G)	12	16.41	64.09 ***	2.60	10.77 ***	0.020	5.52 ***
Environment (E)	1	44.33	173.13 ***	77.80	322.44 ***	0.083	23.24 ***
G × E	12	3.27	12.78 ***	1. 37	5.67 ***	0.007	1.98 *
Error	52	0.26		0.24		0.004	

*, *** stands for 0.05 and 0.001 level of significance, respectively.

**Table 2:** Mean values ± standard error for macro and microelement concentration detected in grain (GR) and grain ash samples (GRash) of the 13 genotypes sown at the two environments (Alto Alentejo-AA and Baixo Alentejo-BA). Different letters represent significant differences between locals for each element.

Macro Concent.	GR Samples		GRash Samples	
	AA (g/kg)	BA (g/kg)	AA (g/kg)	BA (g/kg)
K	8.02 ± 0.16 a	4.94 ± 0.08 b	195.9 ± 3.6 a	110.1 ± 2.2 b
P	1.69 ± 0.04 b	2.06 ± 0.04 a	52.9 ± 1.1 a	52.6 ± 1.1 a
Ca	0.533 ± 0.015 a	0.408 ± 0.009 b	14.3 ± 0.5 a	8.2 ± 0.3 b
S	0.532 ± 0.013 b	0.597 ± 0.006 a	-	-
Cl	0.162 ± 0.010 b	0.243 ± 0.006 a	-	-
Total (%)	99.1	98.6	98.3	97.9
**Micro Concent.**	**AA (mg/kg)**	**BA (mg/kg)**	**AA (g/kg)**	**BA (g/kg)**
Fe	44.0 ± 1.2 a	42.1 ± 0.9 a	1.300 ± 0.035 a	0.789 ± 0.015 b
Mn	35.9 ± 1.1 a	37.1 ± 0.9 a	1.120 ± 0.038 a	0.762 ± 0.019 b
Zn	21.3 ± 0.6 b	30.1 ± 0.5 a	0.727 ± 0.019 a	0.650 ± 0.014 b
Cu	3.49 ± 0.24 b	7.91 ± 0.18 a	0.153 ± 0.005 a	0.124 ± 0.002 b
Si	-	-	0.289 ± 0.057 b	1.106 ± 0.062 a
Rb	-	-	0.956 ± 0.050 a	0.198 ± 0.010 b
Sr	-	-	0.041 ± 0.002 b	0.049 ± 0.002 a
Ti	-	-	0.048 ± 0.002 a	0.031 ± 0.001 b
Total (%)	0.9	1.4	1.7	2.1

**Table 3:** Analysis of variance (F value) for macro- and microelements of 13 genotypes of grain samples (left-GR) and grain ash samples (right-GRash) sown at two different environments (Alto Alentejo and Baixo Alentejo). N = 78.

	Source of Variation							
	GR Samples				GRash Samples			
Element	Model df = 25	G df = 12	E df = 1	G × E df = 12	Model df = 25	G df = 12	E df = 1	G × E df = 12
K	18.79 ***	2.42 *	414.17 ***	2.22 *	30.50 ***	2.64 **	689.68 ***	3.43 **
P	4.48 ***	1.27 ^ns^	60.17 ***	3.04 **	5.14 ***	4.51 ***	0.09 ^ns^	6.19 ***
Ca	13.04 ***	12.69 ***	153.72 ***	1.67 ^ns^	19.12 ***	9.46 ***	333.76 ***	2.56 *
S	2.38 **	1.61 ^ns^	23.86 ***	1.36 ^ns^	-	-	-	-
Cl	6.45 ***	6.44 ***	64.60 ***	1.62 ^ns^	-	-	-	-
Fe	2.72 **	3.06 **	2.39 ^ns^	2.41 *	16.45 ***	4.84 ***	327.25 ***	2.16 *
Mn	4.18 ***	6.71 ***	1.36 ^ns^	1.88 ^ns^	11.30 ***	8.41 ***	159.45 ***	1.84 ^ns^
Zn	9.15 ***	1.97 *	178.17 ***	2.25 *	5.62 ***	5.35 ***	24.53 ***	4.31 ***
Cu	11.82 ***	1.50 ^ns^	256.47 ***	1.76 ^ns^	6.36 ***	6.43 ***	49.67 ***	2.68 **
Si	-	-	-	-	6.74 ***	2.38 *	120.89 ***	1.44 ^ns^
Rb	-	-	-	-	12.44 ***	1.61 ^ns^	270.89 ***	1.74 ^ns^
Sr	-	-	-	-	6.74 ***	10.26 ***	19.26 ***	2.18 *
Ti	-	-	-	-	6.07 ***	4.87 ***	75.10 ***	1.53 ^ns^

^ns^ no significant; *, **, *** stands for 0.05, 0.01, and 0.001 level of significance, respectively.

**Table 4:** Mean values ± standard error for K, P, Fe, and Zn concentration in wheat grain (GR) as influenced by the genotype × environment interaction. Different letters indicate significant differences between the genotype × environment combinations, according to Tukey test (*p* ≤ 0.05).

Genotype	K (g/kg)	P (g/kg)	Fe (mg/kg)	Zn (mg/kg)
AA				
Antalis	8.24 ± 0.14 ab	1.69 ± 0.05 a–d	46.5 ± 2.5 a–c	22.5 ± 2.1 c–f
Arcoduro	8.21 ± 0.29 ab	1.78 ± 0.08 a–d	45.2 ± 2.5 a–c	22.5 ± 1.5 c–f
Aventadur	8.74 ± 0.29 ab	1.60 ± 0.04 cd	47.9 ± 3.3 a–c	20.6 ± 1.5 d–f
Bridão	7.84 ± 0.43 a–c	1.84 ± 0.13 a–d	38.3 ± 3.4 a–c	22.2 ± 1.5 c–f
Celta	7.65 ± 0.64 a–c	1.41 ± 0.08 d	34.9 ± 0.5 bc	19.2 ± 1.5 ef
DNorman	8.84 ± 0.70 ab	1.87 ± 0.11 a–d	46.0 ± 2.5 a–c	20.6 ± 2.1 d–f
DRicardo	7.06 ± 0.14 b–g	1.40 ± 0.08 d	39.5 ± 0.8 a–c	17.3 ± 1.2 f
Fado	9.43 ± 1.33 a	1.88 ± 0.31 a–d	51.8 ± 4.4 a	24.0 ± 3.6 b–f
Gingão	7.33± 0.37 a–e	1.73 ± 0.14 a–d	48.4 ± 2.8 a–c	22.2 ± 0.2 c–f
Kenobi	7.80 ± 0.32 a–c	1.81 ± 0.13 a–d	38.9 ± 2.2 a–c	18.4 ± 1.5 ef
Massimo	8.19 ± 0.30 ab	1.79 ± 0.13 a–d	41.8 ± 1.7 a–c	22.2 ± 0.4 c–f
Trimulato	7.64 ± 0.06 a–d	1.64 ± 0.11 b–d	50.3 ± 4.4 ab	26.0 ± 3.2 a–f
Vadio	7.31 ± 0.25 a–f	1.60 ± 0.08 cd	42.2 ± 2.5 a–c	19.5 ± 1.2 ef
BA				
Antalis	5.20 ± 0.15 f–h	2.21 ± 0.09 a–c	45.0 ± 0.7 a–c	31.8 ± 0.7 ab
Arcoduro	4.82 ± 0.01 h	2.06 ± 0.01 a–d	47.3 ± 1.5 a–c	30.9 ± 0.4 a–c
Aventadur	5.52 ± 0.23 d–h	2.02 ± 0.13 a–d	38.7 ± 2.7 a–c	31.0 ± 2.0 a–c
Bridão	4.85 ± 0.28 h	1.78 ± 0.15 a–d	32.8 ± 1.8 c	27.1 ± 1.7 a–e
Celta	5.88 ± 0.11 c–h	2.29 ± 0.09 ab	43.7 ± 1.6 a–c	33.5 ± 1.4 a
DNorman	5.17 ± 0.14 gh	2.24 ± 0.02 a–c	41.7 ± 1.7 a–c	33.2 ± 0.8 a
DRicardo	5.03 ± 0.09 gh	2.23 ± 0.04 a–c	45.9 ± 2.2 a–c	29.7 ± 1.1 a–d
Fado	4.64 ± 0.25 h	1.83 ± 0.13 a–d	40.1 ± 1.7 a–c	27.1 ± 1.6 a–e
Gingão	4.81 ± 0.17 h	2.06 ± 0.15 a–d	40.2 ± 1.9 a–c	29.5 ± 1.2 a–d
Kenobi	5.26 ± 0.12 e–h	2.34 ± 0.10 a	47.4 ± 2.5 a–c	29.9 ± 0.8 a–c
Massimo	4.17 ± 0.27 h	1.75 ± 0.17 a–d	34.4 ± 3.4 bc	25.5 ± 2.6 a–f
Trimulato	4.30 ± 0.21 h	1.98 ± 0.11 a–d	49.5 ± 3.0 a–c	33.0 ± 1.4 ab
Vadio	4.60 ± 0.10 h	2.05 ± 0.12 a–d	40.6 ± 1.6 a–c	29.0 ± 1.6 a–d

**Table 5:** Mean values ± standard error for Ca, S, Cl, Mn, and Cu concentration in wheat grain (GR) as influenced by the genotype in each experiment (AA—Alto Alentejo; BA—Baixo Alentejo). Diferent capital letters indicate significant diferences in AA according to Tukey test (*p* ≤ 0.05) while lowercase letters indicate significant diferences in BA according to Tukey test (*p* ≤ 0.05).

Genotype	Ca (g/kg)	S (g/kg)	Cl (g/kg)	Mn (mg/kg)	Cu (mg/kg)
AA					
Antalis	0.502 ± 0.032 BC	0.538 ± 0.063 A	0.135 ± 0.021 AB	36.5 ± 4.7 AB	3.23 ± 0.97 A
Arcoduro	0.524 ± 0.028 A–C	0.514 ± 0.036 A	0.104 ± 0.007 AB	36.4 ± 2.6 AB	3.87 ± 0.44 A
Aventadur	0.589 ± 0.005 AB	0.564 ± 0.039 A	0.211 ± 0.049 AB	32.4 ± 2.3 AB	3.60 ± 0.51 A
Bridão	0.493 ± 0.027 BC	0.526 ± 0.037 A	0.137 ± 0.042 AB	37.0 ± 0.7 AB	3.60 ± 1.32 A
Celta	0.604 ± 0.046 AB	0.448 ± 0.036 A	0.231 ± 0.048 A	31.4 ± 0.7 B	1.97 ± 0.47 A
DNorman	0.572 ± 0.035 AB	0.589 ± 0.032 A	0.186 ± 0.029 AB	39.3 ± 4.7 AB	5.23 ± 0.28 A
DRicardo	0.366 ± 0.016 C	0.459 ± 0.037 A	0.036 ± 0.024 B	30.5 ± 1.7 B	2.43 ± 1.39 A
Fado	0.577± 0.081 AB	0.575 ± 0.102 A	0.151 ± 0.037 AB	34.6 ± 3.8 AB	4.17 ± 0.69 A
Gingão	0.700± 0.044 A	0.532 ± 0.031 A	0.264 ± 0.035 A	45.2 ± 3.0 AB	4.70 ± 0.35 A
Kenobi	0.471 ± 0.016 BC	0.527 ± 0.015 A	0.185 ± 0.024 AB	30.8 ± 2.3 B	2.90 ± 1.10 A
Massimo	0.514± 0.006 BC	0.557 ± 0.053 A	0.208 ± 0.040 AB	32.1 ± 3.4 AB	2.80 ± 0.60 A
Trimulato	0.504 ± 0.018 BC	0.607 ± 0.031 A	0.168 ± 0.050 AB	48.1 ± 4.4 A	3.93 ± 0.80 A
Vadio	0.507± 0.017 BC	0.486 ± 0.020 A	0.084 ± 0.028 AB	32.4 ± 2.7 AB	2.97 ± 1.23 A
BA					
Antalis	0.394 ± 0.013 d–f	0.594 ± 0.021 bc	0.229 ± 0.005 cd	37.5 ± 0.6 a–e	8.47 ± 0.80 ab
Arcoduro	0.388 ± 0.005 d–f	0.610 ± 0.011 a–c	0.181 ± 0.007 e	43.4 ± 0.3 ab	8.03 ± 0.26 ab
Aventadur	0.466 ± 0.006 bc	0.554 ± 0.009 c	0.284 ± 0.009 b	33.0 ± 1.5 c–e	7.03 ± 0.24 b
Bridão	0.357 ± 0.014 ef	0.593 ± 0.015 bc	0.265 ± 0.010 bc	31.2 ± 2.8 de	7.87 ± 0.33 ab
Celta	0.490 ±0.002 ab	0.585 ± 0.020 bc	0.253 ± 0.003 b–d	38.7 ± 1.1 a–e	8.00 ± 0.55 ab
DNorman	0.413 ± 0.003 c–e	0.562 ± 0.013 c	0.258 ± 0.019 b–d	37.0 ± 1.1 b–e	8.07 ± 0.23 ab
DRicardo	0.344 ± 0.009 f	0.573 ± 0.018 bc	0.220 ± 0.006 de	36.5 ± 0.7 b–e	8.10 ± 0.70 ab
Fado	0.384 ± 0.006 d–f	0.571 ± 0.004 bc	0.237 ± 0.007 cd	30.6 ± 1.3 de	7.53 ± 0.48 b
Gingão	0.524 ± 0.017 a	0.604 ± 0.025 a–c	0.336 ± 0.005 a	41.7 ± 1.3 a–c	8.27 ± 0.09 ab
Kenobi	0.393 ± 0.008 d–f	0.676 ± 0.005 a	0.218 ± 0.002 de	39.3 ± 1.8 a–d	10.27 ± 0.49 a
Massimo	0.353 ± 0.015 f	0.601 ± 0.003 a–c	0.225 ± 0.003 cd	29.6 ± 3.1 e	7.47 ± 0.88 b
Trimulato	0.359 ± 0.027 ef	0.647 ± 0.005 ab	0.240 ± 0.003 cd	46.3 ± 2.3 a	7.23 ± 0.17 b
Vadio	0.436 ±0.016 b–d	0.594 ± 0.028 bc	0.218 ± 0.011 de	37.0 ± 2.5 b–e	6.57 ± 0.43 b

**Table 6:** Mean values ± standard error for K, P, Ca, Fe, Zn, Cu, and Sr concentration in grain wheat ash as influenced by the genotype × environment interaction. Diferent letters indicate significant diferences between the genotype × environment combinations, according to Tukey test (*p* ≤ 0.05).

Genotype	K (g/kg)	P (g/kg)	Ca (g/kg)	Fe (g/kg)	Zn (g/kg)	Cu (g/kg)	Sr (g/kg)
	AA						
Antalis	196.6 ± 17.5 ab	52.0 ± 4.7 a–c	13.0 ± 1.1 b–f	1.320 ± 0.121 a–d	0.729 ± 0.079 a–d	0.152 ± 0.029 a–f	0.036 ± 0.003 c–f
Arcoduro	191.6 ± 1.1 ab	52.5 ± 0.2 a–c	13.6 ± 0.5 b–e	1.334 ± 0.002 a–d	0.783 ± 0.002 ab	0.176 ± 0.003 a–d	0.040 ± 0.005 c–f
Aventadur	215.8 ± 6.2 a	50.0 ± 2.0 a–d	17.0 ± 0.7 ab	1.343 ± 0.042 a–d	0.737 ± 0.007 a–d	0.133 ± 0.002 b–f	0.051 ± 0.002 a–d
Bridão	215.5 ± 9.0 a	60.4 ± 1.6 ab	14.8 ± 0.3 b–d	1.290 ± 0.057 a–d	0.816 ± 0.043 ab	0.162 ± 0.013 a–e	0.038 ± 0.005 c–f
Celta	182.8 ± 9.7 ab	45.6 ± 2.6 cd	16.0 ± 1.3 a–c	1.064 ± 0.061 c–g	0.662 ± 0.052 b–e	0.126 ± 0.018 d–f	0.052 ± 0.008 a–d
DNorman	200.2 ± 4.1 ab	55.0 ± 1.3 a–c	14.2 ± 0.5 b–e	1.296 ± 0.009 a–d	0.673 ± 0.022 b–e	0.135 ± 0.003 b–f	0.048 ± 0.005 a–e
DRicardo	201.5 ± 9.9 ab	53.6± 3.7 a–c	11.4 ± 0.9 c–h	1.440 ± 0.084 a–c	0.721 ± 0.046 a–d	0.159 ± 0.010 a–e	0.033 ± 0.008 c–f
Fado	181.4 ± 12.7 ab	47.4 ± 4.1 b–d	11.5 ± 1.2 c–h	1.157 ± 0.093 b–e	0.602 ± 0.031 b–e	0.121 ± 0.015 d–f	0.025 ± 0.002 ef
Gingão	185.5 ± 3.6 ab	55.7 ± 1.7 a–c	19.7 ± 1.3 a	1.483 ± 0.007 ab	0.800 ± 0.018 ab	0.184 ± 0.011 a–c	0.066 ± 0.006 ab
Kenobi	211.3 ± 0.6 ab	57.2 ± 1.3 a–c	14.0 ± 0.1 b–e	1.325 ± 0.012 a–d	0.738 ± 0.025 a–d	0.202 ± 0.009 a	0.042 ± 0.003 b–f
Massimo	180.2 ± 9.6 ab	47.4 ± 3.1 b–d	12.5 ± 0.9 b–g	1.085 ± 0.055 c–g	0.660 ± 0.036 b–e	0.127 ± 0.012 c–f	0.035 ± 0.002 c–f
Trimulato	214.6 ± 26.8 ab	63.4 ± 7.0 a	16.0 ± 2.9 a–c	1.658 ± 0.248 a	0.927 ± 0.133 a	0.187 ± 0.022 ab	0.040 ± 0.011 c–f
Vadio	169.6 ± 8.5 bc	48.0 ± 4.7 b–d	11.9 ± 0.7 c–h	1.108 ± 0.050 b–f	0.600 ± 0.043 b–e	0.121 ± 0.009 d–f	0.021 ± 0.003 f
	BA						
Antalis	114.8 ± 0.6 de	57.0 ± 0.9 a–c	8.0 ± 0.2 g–j	0.801 ± 0.003 e–g	0.681 ± 0.008 b–e	0.129 ± 0.001 c–f	0.051 ± 0.002 a–e
Arcoduro	80.8 ± 0.4 e	36.6 ± 0.1 d	5.4 ± 0.1 j	0.744 ± 0.010 fg	0.481 ± 0.005 e	0.101 ± 0.002 f	0.026 ± 0.002 d–f
Aventadur	120.3 ± 6.5 de	48.8 ± 3.4 b–d	9.6 ± 0.6 e–j	0.763 ± 0.034 e–g	0.667 ± 0.042 b–e	0.112 ± 0.006 ef	0.054 ± 0.008 a–c
Bridão	125.6 ± 1.3 c–e	56.4 ± 1.0 a–c	8.4 ± 0.1 f–j	0.721 ± 0.007 fg	0.675 ± 0.011 b–e	0.125 ± 0.002 d–f	0.045 ± 0.002 b–f
Celta	128.7 ± 1.7 cd	56.8 ± 0.8 a–c	10.5 ± 0.5 d–i	0.765 ± 0.018 e–g	0.719 ± 0.019 a–d	0.117 ± 0.003 ef	0.068 ± 0.002 ab
DNorman	107.1 ± 2.8 de	50.9 ± 0.9 a–c	8.1 ± 0.2 g–j	0.707 ± 0.021 g	0.634 ± 0.021 b–e	0.123 ± 0.008 d–f	0.051 ± 0.002 a–e
DRicardo	96.3 ± 0.1 de	46.0 ± 0.0 cd	5.9 ± 0.1 ij	0.732 ± 0.002 fg	0.553 ± 0.006 c–e	0.107 ± 0.001 ef	0.040 ± 0.001 c–f
Fado	115.9 ± 4.9 de	58.1 ± 2.0 a–c	8.5 ± 0.1 f–j	0.830 ± 0.011 e–g	0.667 ± 0.003 b–e	0.118 ± 0.002 ef	0.045 ± 0.002 b–f
Gingão	116.7 ± 1.8 de	57.8 ± 1.4 a–c	11.2 ± 0.1 d–h	0.839 ± 0.014 e–g	0.735 ± 0.012 a–d	0.147 ± 0.002 a–f	0.073 ± 0.008 a
Kenobi	94.2 ± 1.6 de	47.0 ± 1.2 b–d	6.3 ± 0.1 ij	0.719 ± 0.006 fg	0.520 ± 0.007 de	0.143 ± 0.002 b–f	0.039 ± 0.002 c–f
Massimo	117.1 ± 1.6 de	58.0 ± 0.9 a–c	8.5 ± 0.2 f–j	0.799 ± 0.018 e–g	0.683 ± 0.013 b–e	0.130 ± 0.001 c–f	0.053 ± 0.004 a–c
Trimulato	103.2 ±4.0 de	54.4 ± 1.5 a–c	7.4 ± 0.3 h–j	1.040 ± 0.077 d–g	0.760 ± 0.035 a–c	0.144 ± 0.009 b–f	0.049 ± 0.005 a–e
Vadio	109.9 ± 2.3 de	56.5 ± 0.9 a–c	8.6 ± 0.2 f–j	0.798 ± 0.012 e–g	0.669 ± 0.02 b–e	0.116 ± 0.003 ef	0.044 ± 0.003 b–f

**Table 7:** Correlation coefficient of Pearson between ash content and element concentration in grain samples (GR).

	K	P	Ca	S	Cl	Fe	Mn	Zn	Cu
**Ash content**	−0.25 *	0.43 ***	−0.05 ^ns^	0.29 *	0.30 **	−0.04 ^ns^	0.12 ^ns^	0.34 **	0.40 ***

^ns^ no significant; *, **, *** stands for 0.05, 0.01, and 0.001 level of significance, respectively.

## Data Availability

Data are contained within the article and Supplementary Materials.

## Supplementary Materials

The following supporting information can be downloaded at https://www.mdpi.com/article/10.3390/plants14223414/s1, Table S1: Origin, growth habit, and registration year of durum wheat varieties studied; Table S2: Analytical data from soil samples from the locals of the trails: AA—INIAV-Elvas and BA—I.P. Beja—ESA; Table S3: Mean values ± standard error for Mn, Si, Rb, and Ti concentration in grain wheat ash as influenced by the Genotype in each experiment (AA—Alto Alentejo; BA—Baixo Alentejo). Different capital letters indicate significant differences in AA according to Tukey test (*p* ≤ 0.05) while lowercase letters indicate significant differences in BA according to Tukey test (*p* ≤ 0.05).
