# Supplementary material for: Durum Wheat Kernel: Influence of the Genotype and Environment on the Mineral Profile of Grains and Ashes
Source: Plants (Basel). 2025 Nov 7;14(22):3414. doi: 10.3390/plants14223414 (PMC12656325; doi:10.3390/plants14223414)
Supplement: Supplementary file 1 [file plants-14-03414-s001.zip › plants-3874052-supplementary.pdf]

Supplementary Material

Table S1: Origin, growth habit and registration year of durum wheat varieties studied.

Genotype	Origin	Growth habit	Registration year
Massimo Meridio	Italy	Facultative	2010
Antalis	Italy	Facultative/early maturity	2013
Kenobi	Italy	Facultative/ early maturity	2013
Aventadur	Italy	Facultative/early maturity	2016
Arcoduro	Spain	Spring	2005
Don Ricardo	Spain	Spring	2008
Don Norman	Spain	Spring	2012
Trimulato	Spain	Facultative	2012
Celta	Portugal	Spring	1986
Fado	Portugal	Spring	2018
Vadio	Portugal	Spring	2018
Bridão	Portugal	Spring	2021
Gingão	Portugal	Spring	2022

Table S2: Analytical data from soil samples from the locals of the trails: AA - INIAV-Elvas and BA - I.P. Beja - ESA.

Parameters	AA	BA
pH	6.7	8.0
Organic Matter (%)	1.00	1.75
K (mg/kg)	200	109
P (mg/kg)	140	329
Mg (mg/kg)	>220	>220
Fe (mg/kg)	79	44
Mn (mg/kg)	>110	>110
Zn (mg/kg)	1.3	1.5
Cu (mg/kg)	5.7	10.5
B (mg/kg)	0.30	0.55

Table S3: Mean values ± standard error for Mn, Si, Rb and Ti concentration in grain wheat ash as influenced by the Genotype in each experiment (AA-Alto Alentejo; BA-Baixo Alentejo). Different capital letters indicate significant differences in AA according to Tukey test ($p \leq 0.05$) while lowercase letters indicate significant differences in BA according to Tukey test ($p \leq 0.05$).

Genotype	Mn (g/kg)	Si (g/kg)	Rb (g/kg)	Ti (g/kg)
		**AA**		
**Antalis**	1.132±0.120 A-C	0.195±0.022 A	0.912±0.107 A	0.030±0.004 BC
**Arcoduro**	1.099±0.032 A-C	0.161±0.010 A	0.930±0.061 A	0.027±0.009 C
**Aventadur**	1.109±0.050 A-C	0.169±0.119 A	1.290±0.143 A	0.044±0.003 A-C
**Bridão**	1.254±0.111 A-C	0.855±0.298 A	1.239±0.138 A	0.058±0.013 AB
**Celta**	0.934±0.028 BC	0.494±0.242 A	0.662±0.092 A	0.054±0.002 A-C
**DNorman**	1.114±0.027 A-C	0.142±0.036 A	1.000±0.126 A	0.044±0.001 AC
**DRicardo**	1.164±0.072 A-C	0.283±0.061 A	0.869±0.138 A	0.063±0.006 A
**Fado**	0.884±0.088 C	0.110±0.110 A	0.837±0.169 A	0.040±0.001 A-C
**Gingão**	1.426±0.077 AB	0.130±0.123 A	0.949±0.122 A	0.054±0.004 A-C
**Kenobi**	1.089±0.010 A-C	0.137±0.137 A	1.033±0.052 A	0.050±0.002 A-C
**Massimo**	0.962±0.063 BC	0.206±0.119A	0.765±0.078 A	0.055±0.005 A-C
**Trimulato**	1.538±0.266 A	0.328±0.135 A	1.261±0.335 A	0.051±0.008 A-C
**Vadio**	0.853±0.032 C	0.542±0.504 A	0.685±0.127 A	0.050±0.005 A-C
		**BA**		
**Antalis**	0.721±0.006 b-d	1.253±0.128 ab	0.184±0.009 c-g	0.022±0.003 b
**Arcoduro**	0.686±0.008 cd	0.397±0.114 b	0.106±0.001 h	0.029±0.004 ab
**Aventadur**	0.687±0.052 cd	0.921±0.147 ab	0.238±0.027 a-d	0.031±0.004 ab
**Bridão**	0.823±0.010 b	1.191±0.189 ab	0.295±0.014 a	0.038±0.001 ab
**Celta**	0.783±0.023 bc	1.077±0.306 ab	0.224±0.009 a-e	0.044±0.004 a
**DNorman**	0.717±0.028 b-d	1.531±0.191 a	0.175±0.005 d-h	0.032±0.002 ab
**DRicardo**	0.645±0.011 d	0.936±0.139 ab	0.139±0.006 f-h	0.039±0.005 ab
**Fado**	0.732±0.005 b-d	0.962±0.028 ab	0.278±0.032 ab	0.028±0.003 ab
**Gingão**	0.970±0.005 a	1.270±0.245 ab	0.210±0.014 b-f	0.034±0.002 ab
**Kenobi**	0.661±0.008 cd	1.165±0.231 ab	0.153±0.063 e-h	0.025±0.005 b
**Massimo**	0.728±0.014 b-d	0.930±0.059 ab	0.197±0.005 c-f	0.029±0.003 ab
**Trimulato**	1.035±0.061 a	1.184±0.166 ab	0.121±0.007 g-h	0.026±0.003 b
**Vadio**	0.720±0.017 b-d	1.555±0.222 a	0.254±0.006 a-c	0.030±0.001 ab
